# Chia (*Salvia hispanica* L.) a Promising Alternative for Conventional and Gelled Emulsions: Technological and Lipid Structural Characteristics

**DOI:** 10.3390/gels5020019

**Published:** 2019-04-10

**Authors:** Irene Muñoz-González, Esther Merino-Álvarez, Marina Salvador, Tatiana Pintado, Claudia Ruiz-Capillas, Francisco Jiménez-Colmenero, Ana M. Herrero

**Affiliations:** Instituto de Ciencia y Tecnología de Alimentos y Nutrición (ICTAN-CSIC), José Antonio Novais 10, 28040 Madrid, Spain; irene.munoz@csic.es (I.M.-G.); esthermerino14@gmail.com (E.M.-Á.); msalva06@ucm.es (M.S.); tatianap@ictan.csic.es (T.P.); claudia@ictan.csic.es (C.R.-C.); fjimenez@ictan.csic.es (F.J.-C.)

**Keywords:** chia protein, conventional emulsion, emulsion gel, lipid structure, technological properties

## Abstract

Chia (*Salvia hispanica* L.) is an oilseed plant which contains proteins of high biological value and other healthy components with interesting technological properties. For these reasons, chia could be a promising option for the formation and stabilization of oil-in-water emulsions. The aim of this study is to evaluate the potential of chia protein (from chia flour) in the formation of emulsions. To that end, composition and technological and structural properties determined by infrared spectroscopy were investigated in conventional (EC) and gelled (EGC) emulsions with chia and compared with their corresponding soy protein emulsions with the same protein content [conventional (ES) or gelled (EGS)] used as reference. All emulsions containing chia had better fat and water binding properties than those elaborated with soy protein isolate (SPI). The color of the emulsions varied significantly depending on whether the emulsions were made with chia or SPI. EGS and EGC exhibited the greatest (*p* < 0.05) penetration force values, being EGC the firmest (*p* < 0.05). Depending on the type of emulsion, Attenuated Total Reflectance (ATR)-FTIR Spectroscopy revealed differences in their lipid structure and interaction in terms of lipid acyl chain mobility (order/disorder) and emulsion droplet size. These structural characteristics could be related to the textural behavior of emulsions.

## 1. Introduction

In recent years the consumption of vegetable protein as opposed to animal protein has been increasing thanks to concerns about a healthier lifestyle and specific dietary habits. In response to this, a search is under way for alternative sources of vegetable protein capable of providing health benefits and good functional/technological properties. In this context, chia (*Salvia hispanica* L.) is an annual plant of the *Lamiaceae* family and, because of its high oil content, is also considered an oilseed plant native of Mesoamerica which contains proteins of high biological value and offers considerable potential for the development of healthier foods. Chia is gluten-free and contains about 20–25% protein depending on several factors such as climate and crop location [1,2]. Its protein main fraction corresponds to globulins (>50%) with an excellent balance of essential amino acids, especially methionine and cysteine [3,4,5]. High percentages of glutamic acid, arginine and aspartic acid have also been reported in chia protein isolates [4,5,6], being those relevant for the immunologic system and the prevention against heart diseases [7]. In addition, it has likewise been shown that chia (seed or flour) contains proteins and other components such as diverse carbohydrates that form the structure of mucilage which also have interesting emulsifying, gelling and water-fat binding properties [6,8,9]. Moreover, chia has been the focus of study owing to its lipid content, mainly high levels of α-linolenic fatty acid, dietary fiber and other important nutritional components such as vitamins and minerals. It also contains polyphenols making it a promising natural source of antioxidants. All of these components of chia have been associated with a positive and relevant effects on human health [1,2,6,10]. Chia is used for different purposes in several countries (Mexico, Argentina, Chile, New Zealand, Japan, the United States, Canada, and Australia among others). The Dietary Guidelines recommend the consumption of chia as a primary food in amounts not exceeding 15 g/day. In 2009, it was approved as a Novel Food by the European Parliament and European Council [11]. Chia in the human diet is mainly consumed raw in salads, as sprouts or seeds, and added to beverages [12,13]. More recently it is being used as an ingredient in some foods such as cookies, bread, snacks, cake and some meat products [14,15,16,17,18,19]. Chia has also been employed in the preparation of emulsion gels used as animal fat replacers in the development of healthier meat products [20,21,22]. These studies not only show that chia is an alternative source of vegetable protein, but that their properties, i.e., emulsion, gelling and stabilizing capacity, could be a promising option for the formation and stabilization of emulsion gels [20,21,22,23,24].

It would appear that different types of emulsions (conventional, gelled, multiple, etc.) could be formed and stabilized with chia and this would not only improve the nutritional and health properties of emulsions themselves and the products to which these chia emulsions are added, but could also confer good technological properties [6,7,8]. Particularly, it would be desirable to elucidate the potential of chia flour for the formation of different types oil-in-water emulsions. Therefore, the aim of this work was to study chia flour as source of proteins and other compounds (carbohydrates as mucilage, among others) in the formation of both conventional and gelled emulsions. To that end we evaluated the composition benefits and the technological and structural properties of conventional and gelled emulsions elaborated with chia flour. The structural characteristics of emulsions were elucidated using attenuated total reflectance (ATR)-FTIR spectroscopy due to their advantages to provide insights into the behavior of the lipid material at molecular level. The overarching goal was to explore the possibilities as of chia flour as an alternative to develop conventional and gelled emulsion with appropriate technological, structural and nutritional properties. Conventional and gelled emulsions elaborated with soy protein isolated were used as reference.

## 2. Results and Discussion

Table 1 shows the formulation (%) of the different types of oil-in-water emulsions analyzed. In order to facilitate the understanding of their technological and structural properties, the emulsions elaborated with chia flour or soy protein were formulated and prepared with the same final protein concentration, specifically 2.4%. The final protein concentration design for emulsions was achieved considering that soy protein isolate (SPI) contains 79% of protein and chia flour 16%. However, it is necessary to indicate that also in the case of chia flour contains other compounds such as carbohydrates that will also influence these properties of the emulsions. Soy protein emulsions were studied with the aim to use their technological and structural properties as reference since this protein is used habitually in the preparation of emulsions and could be an optimal reference to check if the technological and structural properties of emulsions made with chia are appropriate and viable. These results would be useful to evaluate the potential of chia flour as ingredient to use in the development of conventional or gelled emulsions.

**Figure 1:**
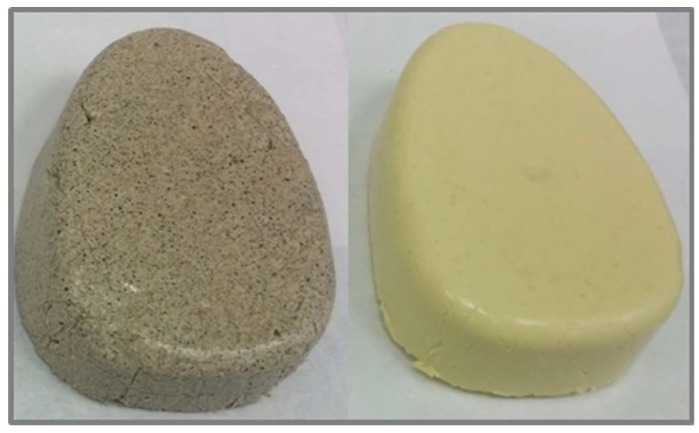
Typical appearance of chia (EGC, **left**) and soy protein (EGS, **right**) emulsion gels. For sample denomination see Table 1.

Two conventional emulsions were prepared, one with 15% chia flour (EC) and another with 3.3% soy protein isolate (ES) (Table 1). Similarly, two emulsion gels were also elaborated using a gelling agent (2%) based on alginate, one of them with chia flour (EGC) and another with soy protein isolate (EGS) as illustrated in Table 1. Figure 1 shows the typical appearance of the soy (EGS) and chia (EGC) protein emulsion gels elaborated.

### 2.1. Proximate Analysis

Table 2 shows the composition of all samples which were generally consistent with product formulation (Table 1). Moisture contents ranged from 43.73 to 57.09% (Table 2) but the significant differences found between each type of emulsion were consistent with the water added in their elaboration (Table 1). Comparisons between similar types of emulsion, conventional or gelled, showed that those containing chia (EC and EGC) had a higher ash content than emulsions with SPI (ES and EGS) owing to the mineral content of chia [25].

Results also showed that ash content was highest (*p* < 0.05) in emulsion gels (EGS and EGC) (Table 2) owing to the added salts used for alginate gelification. Non-significant differences were found in protein content in accordance with the experimental design (Table 1). Protein content was similar (2.4%) for all samples (Table 2) according to the experimental design with the objective of comparing the thermal stability and the technological and structural characteristic of emulsions formulated with soy protein isolate and chia flour as the source of protein, and other compounds such as the different carbohydrates that make up mucilage. Fat content (Table 2) was also close to the target level (40%) but with a different trend between samples depending on the presence of chia flour (Table 1). As for fat content, these emulsions (EC and EGC) had fat from olive oil and chia flour this means a considerable presence of MUFA and n-3 PUFA depending on the fatty acid profile of the olive oil [26], and chia [27]. It is safe to say that chia is a source of healthy lipids, both due to its high α-linolenic acid (ALA) content (62–64%) and its n-6/n-3 (1:3) ratio [9]. According to data provided by the supplier, in conventional and gelled emulsions chia also provides carbohydrates (approximately 0.765 g/100 g) and dietary fiber (approximately 4.5 g/100 g), mainly insoluble, which has been related to intestinal regulation and potential health benefits such as reduction in the risk of breast or colon cancer. Additionally, chia contains various minerals such as calcium, phosphorus, magnesium, potassium and iron, among others [9,10,28].

### 2.2. Thermal Emulsion Stability

The conventional emulsion prepared with soy protein (ES) was the only one which was not thermally stable, exhibiting appreciable phase separation of the emulsion after the analysis. However, it did become thermally stable when the gelling agent (alginate) was added and formed the corresponding emulsion gel (EGS) and only 2% of total loss was observed, 99% of which was water loss. Some authors have evaluated emulsion gels elaborated with different proteins, including soy, stabilized by gelatin, alginate or transglutaminase and reported that none of them formed a bottom aqueous phase indicating that they were more stable due to the incorporation of these gelling agents [23,26,29,30]. All the emulsions containing chia, conventional (EC) and gelled (EGC), exhibited excellent fat and water binding properties with no noticeable (*p* < 0.05) release of exudate after heating. The better thermal stability observed for emulsions containing chia could be due to the interesting technological properties attributed to their different compounds such as proteins and diverse carbohydrates (mainly those composing mucilage) which include high water-holding capacity, water absorption capacity and sufficient emulsifying activity to provide high emulsion stability [6,8,9]. Similar water and fat binding properties have been reported for emulsions prepared with chia, olive oil and different cold-gelling agents [23]. Thanks to the high thermal stability of these olive oil emulsions (conventional and gelled) elaborated with chia, they could be used as animal fat replacers in the formulation of different cooked foods such as healthy lipid-enriched cooked meat products.

### 2.3. Color and pH Measurements

In general, the emulsion gels (EGS and EGC) containing alginate had the lightest color (*p* < 0.05) (Table 3) coinciding with previous results in which emulsion gels containing alginate were lighter than others elaborated with other gelling agents such as transglutaminase or gelatin [23].

Emulsion gel with chia (EGC) had the highest (*p* < 0.05) redness (a*) values (Table 3) which could be attributed to the dark color of this ingredient [2,17]. Conventional or gelled emulsions elaborated with soy protein exhibited higher (*p* < 0.05) b* values than those elaborated with chia (Table 3).

The ES emulsion had the highest pH (7.62) and EC the lowest (6.50), while the emulsion gels had similar (*p* > 0.05) pH values ranging from 6.80 (EGC) to 6.89 (EGS). There were some formulation-dependent differences in the pH values of these EG which, while significant, were of no real practical consequence. Comparable pH values were reported in similar emulsion gels elaborated with soy protein [31] or chia [23].

### 2.4. Textural Properties

The penetration test results (Table 3) differentiated various types (*p* < 0.05) of textural behavior in olive oil emulsions depending on their formulation (Table 1). In sample ES, plunger penetration did not produce a breaking point and this emulsion did not present puncture force (PF) or gel strength (GS) values. In this case, the system behaved like a viscous material lacking a gel structure. The other emulsions behaved quite differently (Table 3), with plunger penetration producing a breaking point characteristic of a gel structure. Of these emulsions, those containing alginate (EGS and EGC) presented the highest (*p* < 0.05) PF and GS values (Table 3). It has previously been described that emulsion gels containing alginate exhibit stronger gel strength [23] which could be attributed to smaller emulsion droplets and a main structure of particle-filled biopolymer gel [32]. Alginate gels consist of cross-linked polymeric molecules that form a three-dimensional macro-molecular network containing a large water fraction in the structure and are mechanically rigid [33]. A comparison of emulsion gels showed that the samples containing chia (EGC) had higher (*p* < 0.05) PF and GS than soy protein samples (EGS) which could be due to chia also containing certain compounds such as mucilage (from part of the carbohydrates found in chia flour) with gelling properties [8,9,34] which could contribute to the higher PF and GS of EGC (Table 2). Additionally, chia protein aid gel formation in certain conditions [6,34,35] which can also lead to higher PF and GS (Table 3). These chia protein and mucilage properties could also be responsible for the different textural behavior observed in conventional emulsions of soy (ES) and chia (EC) since only the ones containing chia (EC) exhibited a gel behavior (Table 3).

### 2.5. Structural Analysis

#### 2.5.1. Lipid Structure

**Figure 2:**
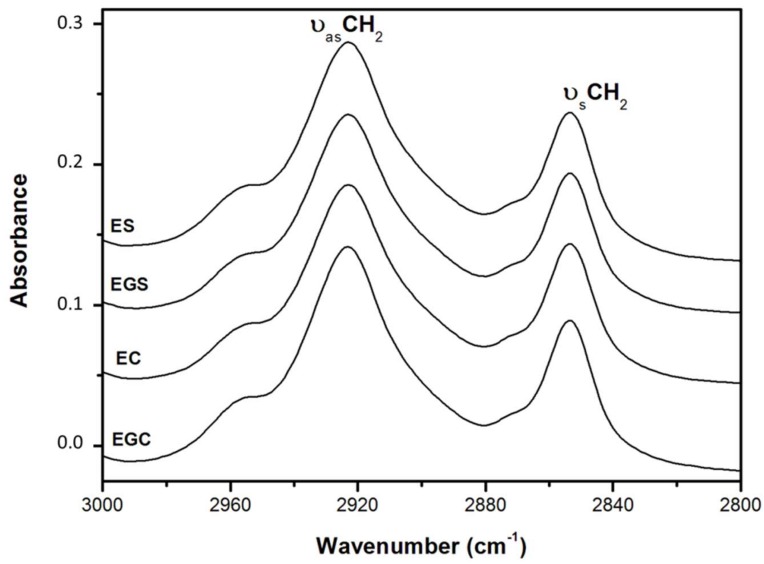
ATR-FTIR spectra in the 2980–2820 cm^−1^ region of conventional emulsions and emulsion gels. For sample denomination see Table 1.

Figure 2 shows the typical ATR-FTIR spectra in the 2980–2820 cm^−1^ region of the different conventional and gelled emulsions. This spectral region shows some characteristic lipid functional groups and is dominated by two strong bands at about 2923 and 2854 cm^−1^. These bands are the result, respectively, of the asymmetric and symmetric stretching vibrations of the acyl CH_2_ groups [29,36,37].

Alterations of these infrared bands in terms of an increase in half-bandwidth or broadening are generally attributed to changes in the conformational order of the lipid acyl chains and to their dynamics [29,37,38]. The interaction of lipids with other biomolecules such as proteins normally generates spectral changes in the methylene νCH modes of lipid chains, generally more pronounced in the asymmetric bands (ν_as_CH_2_) at 2923 cm^−1^ than in symmetric (ν_s_CH_2_) bands at 2854 cm^−1^ [39]. In this respect, non-significant half-bandwidth changes were observed in the symmetric (ν_s_CH_2_) bands at 2854 cm^−1^ (Table 4). However, spectral results showed a significant increase or broadening of the ν_as_CH_2_ at 2923 cm^−1^ when compared to olive oil with conventional emulsions or emulsion gels (Table 4). This increase (*p* < 0.05) in half-bandwidth may be related to the fact that the oil acyl chains are involved in interactions between structurally dissimilar acyl and protein chains as well as in other interactions such as intermolecular contacts between oil acyl chains and water and/or carbohydrate molecules [29,37]. These lipid interactions with other molecules (protein, water, etc.) could cause disorder in the olive oil lipid chain in conventional emulsions or emulsion gel formation. The broadening of the ν_as_CH_2_ band at 2923 cm^−1^ was the highest (*p* < 0.05) in ES and EC samples independently of the use soy protein isolate or chia in their preparation. This structural characteristic (Table 4) could be related with the different textural behavior observed when compared to conventional emulsions or emulsion gels which showed higher PF and GS (Table 3). Oil-in-water emulsions elaborated with caseinate and soy protein and their corresponding emulsion gels prepared with transglutaminase as a gelling agent showed the lowest broadening of the ν_as_CH_2_ band, related to the greatest lipid chain order and hence the greatest firmness [29,37]. It could be that the protein source and the gelling agent provide specific structural and textural characteristics of the emulsions.

#### 2.5.2. OH Stretching Band

**Figure 3:**
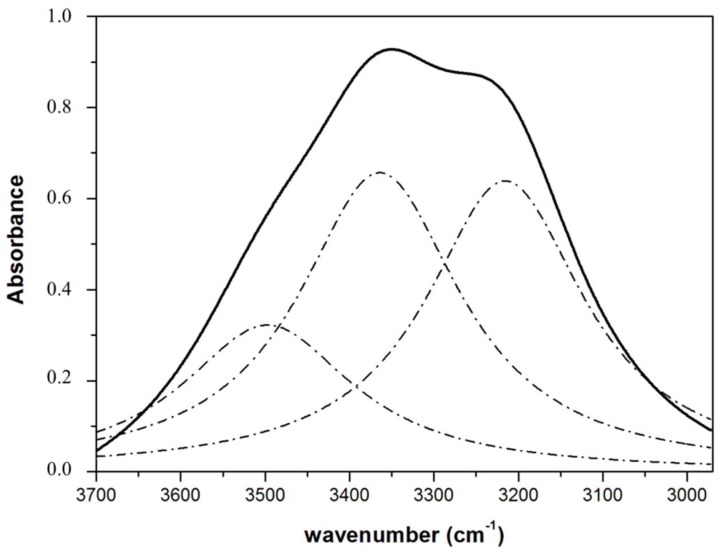
Typical spectrum of soy protein emulsion gel (EGS) and the Lorentzian OH stretching band components in the 3700–2950 cm^−1^ region. For sample denomination see Table 1.

A broad absorption band in the 3600–3100 cm^−1^ region corresponds to the characteristic O–H stretching vibration and hydrogen bond of the hydroxyl groups. Figure 3 shows the O–H stretch region for a typical spectrum of soy protein emulsion gel (EGS) in this spectral region and the Lorentzian OH stretching band components.

In general, the spectrum of pure water and all conventional emulsions and emulsion gels shows a good fit in terms of three Lorentzian components centered near 3592, 3382 and 3216 cm^−1^. The relative band area around 3216 cm^−1^ has been associated with the measure of collectiveness of vibrations within the fraction of water molecules that take part in the tetrahedral structure [23]. Quantification of the relative band area around 3220 cm^−1^ showed a significant decrease respect of olive oil due to the formation of conventional emulsion or emulsion gel, being more significant in emulsion gel EGS and EGC (Table 4). This area decreasing indicates a weakening of the hydrogen bond of the tetrahedrally coordinated water structure that was more relevant (*p* < 0.05) in the emulsion gels, particularly more significant in those elaborated with chia. Similar results were previously observed in chia emulsion gels prepared with various gelling agents [23]. Additionally, the characteristic OH stretching band exhibits significant changes when water core size is decreased [40]. Therefore, the lower (*p* < 0.05) relative area band values of the 3216 cm^−1^ band for emulsion gels, EGS and EGC, compared with conventional emulsions, ES and EC, (Table 4) can be explained in terms of lower (*p* > 0.05) emulsion droplet size in the latter [23]. This is related to the decrease of the hydro-dynamic radii (*Rh*) of emulsion droplets [40], implying that the size of the emulsion droplet incorporated into the three dimensional structure of emulsion gels is smaller than in conventional emulsions. Of the different emulsion gels, this behavior was more significant in the emulsion gel prepared with chia (EGC) (Table 4). It has been shown that oil droplet size has important effects on the rheological properties of emulsion gels [41,42]. In general, it would appear that emulsion gels containing small oil droplets are characterized by higher compressive stress, strain and energy values and gel strength than those of emulsion gels containing large oil emulsion droplets [42,43]. Some authors have also described how small droplets can fit into the gel-network and act as anchor points which increase gel strength [42]. Based on these studies, the results of the OH stretching band area related to emulsion droplet size seem to be consistent with the differences found in the textural behavior of EGS and EGC emulsion gels (Table 3). These emulsion gels were characterized by lower relative OH band area (Table 4) associated with smaller emulsion droplet size and higher PF and GS (Table 3). These textural properties are expected because their smaller droplet size can be incorporated into the network, reinforce its structure and consequently increase gel strength [42,44], being this more relevant in the case of emulsion gel elaborated with chia perhaps due to its properties, mainly in terms of emulsion and gelling capacity described for their proteins and mucilage components (formed by chia flour carbohydrates) [6,8,9,34].

## 3. Conclusions

This research highlights the possible use of chia flour as an alternative due to proteins and other compounds such as mucilage for the elaboration of conventional and gelled oil-in-water emulsions with healthy composition and suitable technological properties. The inclusion of chia flour in conventional and gelled emulsion formation provides good thermal stability and appropriate technological properties such as texture and water and fat binding properties characteristics, being emulsion gels characterized by the greatest thermal stability and firmness. Regarding the structural characteristics of lipids, the extent of lipid chain disorder or lipid interactions depends on the type of emulsion (conventional or gelled) given that emulsion gels, regardless of whether was from soy or chia, exhibited the greatest lipid chain order when compared to conventional emulsions. Additionally, emulsion gels were characterized by lower droplet size, a fact which was more relevant when chia was part of the emulsion gel. All of this would appear to indicate that there may be a relationship between structural and textural behavior observed in all emulsions.

## 4. Materials and Methods

### 4.1. Materials

The ingredients used to make oil-in-water conventional emulsions and emulsion gels were: soy protein isolate (SPI) (approximately ≥ 79% protein content, and ≤ 1% fat) (Vicoprot, TRADES S.A., Barcelona, Spain); chia flour (Salvia hispanica L.) (approximately 16% protein, 31% fat and 30% dietary fiber, total carbohydrate 5.1 g/100 g and ash 4.4 g/100 g according to the information provided by the supplier) (Primaria Premium Raw Materials S.L., Valencia, Spain); sodium alginate (Tradissimo, TRADES S.A., Barcelona, Spain); calcium sulphate 2-hydrate (Panreac Química S.A., Madrid, Spain), tetra-sodium pyrophosphate 10-hydrate (Panreac Química S.A., Madrid, Spain) and olive oil (Carbonell Virgen Extra, SOS Cuétara S.A., Madrid, Spain).

### 4.2. Preparation of Olive Oil-in-Water Conventional Emulsions and Emulsion Gels

Conventional emulsions (ES and EC) were prepared by mixing the corresponding amounts of water and soy protein isolate (SPI) or chia flour (C) (Table 1) for 30 s in a homogenizer (Thermomix TM 31, Vorwerk España M.S.L., S.C., Madrid, Spain) at 5600 rpm approx. Olive oil (Table 1) was then gradually added while the homogenizer was running (approx. 5600 rpm). Emulsion gels (EGS and EGC) were elaborated according to Pintado et al. (2015) [23] by mixing the corresponding amounts of water and soy protein isolate (SPI) or chia flour (C) (Table 1) for 30 s in the homogenizer (approx. 5600 rpm), and then the alginate-based gelling agent was added and mixed (15 s at approximately 5600 rpm). Olive oil was then gradually added to the mixture while the homogenizer was running (5600 rpm).

All the emulsions were then placed in a metal container under pressure to compact them and prevent air bubbles and stored in a chilled room at 2 °C for 20 h. The metal containers were then removed and the emulsions were analyzed. Each type of sample (700 g) was prepared in triplicate.

### 4.3. Proximate Analysis

Sample moisture and ash contents were determined (AOAC, 2000) in triplicate. Protein content was measured in quadruplicate with a LECO FP-2000 Nitrogen Determinator (Leco Corporation, St Joseph, MI, USA). Fat content was evaluated in triplicate according to Bligh and Dyer (1959) [45].

### 4.4. Thermal Emulsion Stability

The thermal stability of samples water and fat binding was determined (in triplicate) based on the procedure of Jiménez-Colmenero, Carballo, & Solas (1995) [46]. The samples (approx. 15 g) were compressed into hermetically sealed tubes, centrifuged (3500 rpm, 5 min) and heated in a water bath for 30 min at 70 °C. Once the heating process was concluded, they were opened and left to stand upside down (for 50 min) to release the separated fat and water onto a plate. Total loss was measured as % of initial sample weight. Water loss was determined as % weight loss after heating the total released fluid (fat and water) for 16 h on a stove at 100 °C. Fat loss was calculated as the difference between total and water losses.

### 4.5. Color and pH Measurements

Color (CIE-LAB tristimulus values, lightness, L*; redness, a* and yellowness, b*) was evaluated on a Chroma Meter CM-3500d (Konica Minolta Business Technologies, Inc., Tokyo, Japan). Determinations were carried out on cross-sections of sample. Ten determinations were performed for each emulsion.

pH was measured in triplicate using an 827 Metrohm pH-meter (MetrohmAG, Herisau, Switzerland) on homogenates of sample in distilled water at a ratio of 1:10 *w/v*.

### 4.6. Textural Properties

Penetration tests were performed at approximately 22 °C using a TA-XT plus Texture Analyzer (Texture Technologies Corp., Scarsdale, NY, USA) and the Texture Exponent program. A load cell of 5 kg was employed. The penetration test was performed according to Herrero et al. (2011a) [29]. Analysis was conducted with a 4 mm diameter cylindrical stainless steel plunger at a velocity of 0.8 mm/s and force exerted at 10 mm. The textural parameters of each sample derived from their force-deformation curves were: (a) penetration force (PF, N) at the point of gel rupture and (b) gel strength (GS, Nmm), which is defined as the area enclosed by the force-deformation curve at the point of gel rupture. Five measurements per sample were performed.

### 4.7. Lipid Structural Characteristics

#### 4.7.1. Attenuated Total Reflectance (ATR)-FTIR Spectroscopy

##### Spectroscopic Measurements

The same mixtures and contents of soy protein and chia flour, with and without a gelling agent in aqueous solution (without added olive oil), were prepared as references for spectroscopic measurements. 

The infrared spectra of each sample (conventional emulsions and emulsion gels and their corresponding references) were recorded using a Perkin-Elmer Spectrum TM 400 spectrometer (Perkin Elmer Inc., Tres Cantos, Madrid, Spain) in mid-IR mode, equipped with an ATR (attenuated total reflectance) sampling device containing diamond/ZnSe crystal. Measurements were performed at room temperature using approximately 25 mg of the samples (without any prior sample preparation), which were placed on the surface of the ATR crystal and slightly pressed with a flat-tip plunger. The spectra were scanned in the 4000–650 cm^−1^ wave number range with a scan speed of 0.20 cm/s and 8 accumulations at a resolution of 4 cm^−1^. Nine measurements of each sample were taken. Sums of three spectra (24 accumulations) were performed. Three sum spectra were analyzed for each type of O/W emulsion gel. A background spectrum was scanned under the same instrumental conditions before each series of measurements.

##### Data Analysis

Spectra were acquired with the Spectrum software version 6.3.2 and spectral data were processed with the Grams/AI version 9.1 (Thermo Electron Corporation, Waltham, MA, USA) software.

The spectral region 3000–2800 cm^−1^ was analyzed to study the lipid structural characteristics of conventional emulsions (ES and EC) and emulsion gels (EGS and EGC). To avoid the spectral influence of water or of any other ingredients in this spectral region, their contribution was subtracted using the appropriate spectra of each sample aqueous solution without olive oil elaborated as a reference by applying the 2125 cm^−1^ association band of water as an internal intensity standard [23,47,48]. To eliminate any spectral influence of proteins in this spectral region (3000–2800 cm^−1^), the resulting spectra were then subtracted using a subtraction factor to eliminate the amide II band such that the maximum intensity near 1545 cm^−1^ was not visible. The half-bandwidth of the 2923 (ν_as_CH_2_) and 2854 (ν_s_CH_2_) cm^−1^ bands were measured in the resulting difference in spectra. Lorentzian OH stretching band components in the 3700–2950 cm^−1^ region were also calculated and then the relative band area around 3216 cm^−1^ was quantified.

### 4.8. Statistical Analysis

One-way analysis of variance (ANOVA) was performed to evaluate the statistical significance (*p* < 0.05) of the effect of different types of emulsion formulations using the SPSS program (v.22, IBM SPSS Inc.; Chicago, IL, USA). Least squares differences were used for comparison of mean values among formulations and Tukey’s HSD test was used to identify significant differences (*p* < 0.05) between formulations.

## Figures and Tables

**Table 1:** Formulation (%) of olive oil conventional emulsions and emulsion gels.

Samples ^2^	SPI ^1^	Chia Flour	Water	Olive Oil	Gelling Agent		
					Sodium Alginate	CaSO_4_	Sodium Pyrophosphate
ES	3.3		56.7	40			
EC		15.0	45.0	40			
EGS	3.3		54.7	40	0.73	0.73	0.54
EGC		15.0	43.0	40	0.73	0.73	0.54

^1^ soy protein isolate (SPI). ^2^ Conventional emulsions prepared with olive oil, water and soy protein isolate (ES) or chia flour (EC), and emulsion gels elaborated with olive oil, water and a gelling agent based on alginate, with soy protein isolate (EGS) or chia flour (EGC).

**Table 2:** Proximate analysis (%) of olive oil conventional emulsions and emulsion gels.

Samples ^1^	Moisture	Ash	Protein	Fat
ES	57.09 ± 0.43 ^a^	0.17 ± 0.01 ^d^	2.40 ± 0.19 ^a^	37.31 ± 1.57 ^a^
EC	45.54 ± 0.16 ^c^	0.64 ± 0.05 ^c^	2.41 ± 0.01 ^a^	38.93 ± 1.05 ^a^
EGS	54.65 ± 0.51 ^b^	1.04 ± 0.01 ^b^	2.42 ± 0.16 ^a^	38.19 ± 1.26 ^a^
EGC	43.73 ± 0.15 ^d^	1.55 ± 0.05 ^a^	2.39 ± 0.02 ^a^	40.69 ± 1.20 ^a^

^1^ For sample denomination see Table 1. Means ± standard deviation. Different letters (a, b, c and d) in the same column indicate significant differences (*p* < 0.05).

**Table 3:** Color [(L*) lightness, (a*) redness and (b*) yellowness)] and texture parameters [puncture force (PF, N) and gel strength (GS, Nmm)] of olive oil conventional emulsions and emulsion gels.

Samples ^1^	L*	a*	b*	PF	GS
ES	57.16 ± 0.29 ^d^	−0.15 ± 0.06 ^d^	22.94 ± 0.44 ^b^	Nd ^**^	Nd ^**^
EC	61.72 ± 1.00 ^c^	0.10 ± 0.03 ^c^	9.51 ± 0.44 ^d^	0.13 ± 0.01 ^c^	0.25 ± 0.02 ^c^
EGS	76.62 ± 0.47 ^a^	0.38 ± 0.04 ^b^	26.14 ± 0.20 ^a^	0.34 ± 0.02 ^b^	0.79 ± 0.07 ^b^
EGC	64.56 ± 0.97 ^b^	1.69 ± 0.14 ^a^	11.47 ± 0.39 ^c^	1.43 ± 0.07 ^a^	3.12 ± 0.40 ^a^

^1^ For sample denomination see Table 1. Means ± standard deviation. Different letters (a, b, c and d) in the same column indicate significant differences (*p* < 0.05). Nd **: Not detected.

**Table 4:** Half-bandwidth values of the 2923 cm^−1^ (ν_as_CH_2_) and 2854 cm^−1^ (ν_s_CH_2_) bands of pure olive oil and conventional emulsions and emulsion gels, and relative area values of the OH stretching band (3220 cm^−1^) of water and conventional emulsions and emulsion gels.

Samples ^1^	Half-Bandwidth 2923 cm^−1^ (ν_as_CH_2_)	Half-Bandwidth 2854 cm^−1^ (ν_s_CH_2_)	Area 3220 cm^−1^ Band
Olive oil	27.5 ± 0.1 ^c^	16.2 ± 0.2 ^a^	-
Pure water	-	-	0.427 ± 0.004 ^a^
ES	30.1 ± 0.2 ^a^	16.5 ± 0.3 ^a^	0.382 ± 0.002 ^b^
EC	31.3 ± 0.3 ^a^	16.5 ± 0.5 ^a^	0.379 ± 0.004 ^b^
EGS	28.9 ± 0.1 ^b^	16.9 ± 0.2 ^a^	0.365 ± 0.001 ^c^
EGC	29.3 ± 0.2 ^b^	16.4 ± 0.4 ^a^	0.343 ± 0.001 ^d^

^1^ For sample denomination see Table 1. Means ± standard deviation. Different letters (a, b, c and d) in the same column indicate significant differences (*p* < 0.05).
